# Effects of warm needle acupuncture on behavioral pain threshold in a rat model of migraine

**DOI:** 10.3389/fneur.2025.1538182

**Published:** 2025-10-01

**Authors:** Qiang Liu, Xiaoguang Qin, Chenglin Luo, Zhongting Zhao, Wangjun Xie, Xiaozheng Du, Xin Qin

**Affiliations:** ^1^Department of Acupuncture and Moxibustion, Gansu University of Traditional Chinese Medicine, Lanzhou, China; ^2^Medical Faculty, Northwest Minzu University, Lanzhou, China

**Keywords:** needle warming acupuncture, migraine, ET-1 protein, 5-HT, CGRP

## Abstract

**Background:**

As a traditional Chinese treatment method, acupuncture has a long history in the treatment of migraine and a rich literature base. As a traditional acupuncture technique, warm needle acupuncture has clearly demonstrated clinical efficacy in the treatment of migraine, although its mechanism of action remains unclear. This study aimed to evaluate the efficacy and safety of warm needle acupuncture in the treatment of migraine in a rat model of migraine by comparing pain threshold and analgesic-related factors among different groups.

**Methods:**

Thirty Sprague–Dawley rats were randomly divided into control, model, and acupuncture groups, with 10 animals in each group. On day 8, rat migraine models were established by subcutaneous injection of nitroglycerin. The acupuncture group was bilaterally needled once per day at Fengchi, Baihui, Fengfu, and Hegu. Behavioral changes and biochemical alterations among the rats before and after modeling were observed for 7 consecutive days after treatment. The expression of Endothelin-1 (ET-1), interleukin (IL)-6 and tumor necrosis factor-alpha (TNF-*α*) in the brain stem of the animals were assessed using ELISA. Expression levels of calcitonin gene-related peptide (CGRP) mRNA were determined using real-time quantitative polymerase chain reaction techniques.

**Results:**

After treatment, the expression of ET-1, IL-6, TNF-*α*, and CGRP in the brain stem of the model group was significantly increased compared with that of the control group (*p* < 0.05), while the expression of IL-6, TNF-*α*, and CGRP in the brain stem of the acupuncture group was decreased.

**Conclusion:**

Warm needle acupuncture reduced ET-1 protein levels and promoted the expression of neurotransmitters, such as 5-hydroxytryptamine, in migraine rat model animals. In addition, needle warming acupuncture decreased IL-6, TNF-*α*, and CGRP levels in the brain stem of the migraine group, which may explain its mechanism of action in treating migraine.

## Introduction

Migraine is a common, multifactorial neurovascular disorder characterized by recurrent attacks, with mild to severe one- or two-sided episodic, pulsatile headaches, and a variety of botanic neurological symptoms, such as nausea, vomiting, photophobia, and phobias (1, 2). The incidence of migraines is high. Migraine is associated with thromboxins and vasoactive substances that increase the risk of ischemic stroke and cardiovascular disease. In addition, 34–57% of migraines are accompanied by depression (3). Migraines are experienced by 10–13% of the population in the United States, with approximately three times as many women as men (4). Because the pathogenesis of migraine(s) remains unclear, the abuse of painkillers can cause chronic daily headaches and drug-reversal headaches, thus making the treatment of migraine a major clinical problem (5, 6).

Experimental animal models can simulate the clinical manifestations and pathogenesis of human diseases, and can be replicated and collected in large quantities to help reduce the risk of subsequent clinical trials. Nitroglycerin, a donor of the fat-soluble neurotransmitter nitric oxide (NO), easily passes the blood–brain barrier and can directly stimulate vascular smooth muscle, leading to vascular dilation. In migraine patients during the attack, peripheral blood NO and its metabolite nitrite will increase. Previous studies have shown that NO produced by nitroglycerin can directly activate the second messenger cyclic guanosine monophosphate (cGMP), and induce the expansion of intracranial and external blood vessels through regulation of downstream signaling pathways, activate nociceptive neurons, and induce the onset of migraine. In addition, NO can also promote the release of CGRP from the trigeminal nerve endings around the cerebrovascular, causing neurogenic inflammatory response and cerebral vascular overexpansion, thus participating in migraine.

Although current anti-migraine drugs are effective, their safety, tolerability, and long-term efficacy are unsatisfactory (7). Acupuncture in the treatment of migraine has the characteristics of a precise curative effect and few adverse reactions and can effectively reduce the frequency of migraine attacks, relieve the degree of pain, prevent recurrence, and improve patient quality of life (8, 9). Acupuncture has a long history in China, which was recorded in detail as early as the “Huangdi Neijing,” and is an important part of Chinese medicine. As an effective treatment with few side effects, acupuncture has many indications and has been widely used in the treatment of a variety of functional disorders, such as the treatment of various painful diseases and acupuncture anesthesia. Acupuncture treatment for pain is one of the important characteristics of traditional Chinese medicine. The analgesic effect induced by acupuncture has been used in clinic for a long time. At present, a variety of acupuncture analgesia methods have been derived on the basis of traditional acupuncture analgesia. Among them, warm needle acupuncture has been applied in the treatment of many diseases.

Elevated levels of calcitonin gene-related peptide (CGRP), interleukin (IL)-6, and tumor necrosis factor-*α* (TNF-α) in migraine (10, 11). The brain stem is the transit station of nociceptive stimulation. When peripheral nociceptive stimulation travels along the central process of the trigeminal nerve to the nucleus spinalis fascicularis of the trigeminal nerve nucleus (STN), it also stimulates the endogenous analgesic system of the brain stem. IL-6 and TNF-α are potential pain mediators, in which IL-6 can enhance the excitability of the meningeal afferent system and promote pain signaling, thus participating in the development of migraine. The increased release of CGRP is related to the activation of trigeminal ganglion, which is also an important reason for promoting the transmission of pain information, stimulating the cerebrovascular wall and causing throb headache (10, 11). In addition, the occurrence of migraine has a documented relationship with dopamine, 5-hydroxytryptamine (5-HT), and other neurotransmitters (12, 13). The 5-HT is one of the autologous active substances, mediating the regulation of pain, sleep and body temperature and other physiological functions. The increase of its expression will promote vasospasm and thus participate in the development of migraine (12, 13). Biomedical approaches are more common in the clinical treatment of migraine, including ergot preparations and triptans are used frequently (14). However, the therapeutic effects of these preparations varies greatly. The therapeutic outcomes of these specific medications used here vary and often the symptoms relapse (15).

As a therapeutic approach, warm needle acupuncture and moxibustion can unclog veins, promote blood circulation, and remove blood stasis, which can be used for the treatment of various diseases, including migraines (16, 17). Warm needle acupuncture is used at fixed acupoints or other anatomical locations to provide warm acupuncture moxibustion stimulation to prevent and treat diseases (18). Warm needle acupuncture can improve blood circulation, improve immune function, and stimulate and enhance the body’s resistance to disease (19). In addition, it exerts anti-inflammatory, analgesic, and regulatory effects on the nervous and endocrine systems (20). However, the mechanism of action of warm needle acupuncture in migraine remains unclear. As such, the effects of warm needle acupuncture on the levels of IL-6, TNF-*α*, 5-HT, CGRP, and ET-1 proteins in a nitroglycerin-induced migraine rat model were examined to explore the possible mechanism of action.

## Methods

### Establishment of rat migraine model

Thirty four-month-old male Sprague–Dawley rats were randomly divided into three groups (*n* = 10 each). Except for the control group, migraine model animals were established using subcutaneous injection of nitroglycerin (10 mg/kg) into the posterior neck of the other two groups. Procedures involving animals in this study were conducted in accordance with the Declaration of Helsinki. All animal experiments were performed in accordance with the relevant guidelines and regulations, and were approved by Gansu University of Traditional Chinese Medicine (Approval code: 2020-100). All animal procedures were performed in accordance with the Guide for the Care and Use of Laboratory Animals (US and National Institutes of Health). Caudal artery blood was collected, and the serum was separated and stored at −80 °C. After blood collection, rats from each group were euthanized in a CO_2_ chamber. The brain tissue of the rats was removed, and the brain stem was separated into two parts along the posterior midline. The trigeminal spinal tract nucleus on one side of the brainstem was removed. The trigeminal spinal tract nucleus of the brain stem was rinsed with ice-cold normal saline, frozen in liquid nitrogen, and stored at −80 °C for future use.

### Treatment measures

All the rats were fed a normal diet. The rats in the model group and the acupuncture group were fed for 7 days and were injected subcutaneously in the posterior neck with nitroglycerin (10 mg/kg) starting from day 8. In addition, on day 8, the animals of the acupuncture group also were immobilized, fixed, and treated using a warm needle. Electroacupuncture integrated instrument (HT-2WN) was purchased from Beijing Huaxi Technology Company, and Huatuo brand silver needles were purchased from Jieyang Jiaxing Medical Equipment Co., LTD. Briefly, before the acupuncture, animals were held and their eyes covered to soothe and reduce tension. The selected acupoints of rats included: Fengchi, Baihui, Fengfu and Hegu. The bilateral Fengchi points were located at the depression between the sternocleidomastoid and trapezius muscles, approximately 3 mm from the center of the line between the ears, under the occipital bone of the posterior neck; Baihui point, the middle of the parietal bone, oblique stab forward or backward 2 mm; Fengfu point, occipital crest posterior occipital atlas joint dorsal depression, oblique stab 1 mm backward and downward; Hegu point, between the first and second metacarpal of the forelimb, straight stab 4 mm. Acupoint warm acupuncture treatment: the electroacupuncture apparatus was used after acupuncture by Huatuo brand 0.25 mm × 13 mm millimeter needle at a temperature of 45 °C and a current of 1 mA. The stimulation time was 10 min/time.

### Facial allodynia

The rats were placed in a custom-made plastic tube restraint with barbed wire in front to ensure access to the periorbital area. After an adaptation period of 30 min, the von-Frey tip was applied to the periorbital area with steady vertical pressure until a forceful stroke of the face or head with the ipsilateral front paw quickly retreated from stimulation or sound, which could be judged as a positive withdrawal response.

### Hind-paw allodynia

Before the test, the rats were placed individually on a dedicated elevated test rack for 30 min to acclimate them to the arena. The elevated test frame consists of a row of 12 transparent plexiglass housings separated by opaque partitions with wire mesh at the bottom. Mechanical stimulation is applied intermittently until the claw is withdrawn from the tip or lifted from the mesh floor. Repeat 3 times on each side, 30 s between each stimulation. The average of 3 (face)/6 (rear paw) measurements is considered the threshold, with a critical value of 60 g.

### RNA extraction and real-time quantitative polymerase chain reaction

The trigeminal spinal tract nucleus was removed from the brain tissue of the rats. Total trigeminal spinal tract nucleus RNAs were isolated using TRIzol reagent (Invitrogen, Waltham, MA, United States) according to manufacturer’s instructions. First-strand complementary DNA was synthesized using a commercially available first strand synthesis kit (Takara, Dalian, China), and real-time quantitative polymerase chain reaction (RT-qPCR) was performed using gene-specific primers and the SYBR premix EX TAQ II kit (RR820A, Takara, Dalian, China), as described previously. The following PCR conditions were used: denaturation at 95 °C for 10 min; 45 amplification cycles, with denaturation at 95 °C for 15 s, annealing and extension at 60 °C for 1 min. RT-qPCR was performed using a real-time PCR thermocycler system (7500, Applied Biosystems, Carlsbad, CA, United States). The 2^−∆∆Ct^ method was used to analyze relative expression levels, and GAPDH was used as the internal reference gene. RT-qPCR primers used in this study were CGRP-Forward (F): 5′–CTGGTAGCAAATCTAGTCCCCA–3′ and CGRP-Reverse (R), 5′–CCATGTGTCCCCAGAAGACCAAG–3′; GAPDH-F: 5′–AGCCAAAAGGGTCATCATCT–3′ and GAPDH-R: 5′–GGGGCCATCCACAGTCTTCT–3′.

### ELISA

Commercially available ELISA kits were purchased from Elabscience Biotechnology Co., Ltd. (Wuhan, China) to determine the levels of ET-1, IL-6, and TNF-*α* in serum. All assays were performed in accordance with manufacturer’s instructions. Each sample was analyzed three times independently.

Levels of 5-HT in brain tissue.

### Levels of 5-HT in brain tissue

The brain stem tissue homogenized pulp was prepared, the supernatant (3,000 r/min, 15 min) was centrifuged, and the 5-HT level in the tissue was detected by ELISA kit. Follow the kit instructions and repeat three times for each sample.

### Western blot

Proteins from tissues or cells were extracted and separated using 10% sodium dodecyl sulfate-polyacrylamide gel electrophoresis. The protein concentrations in the supernatants were quantified using a Micro BCA Protein Assay Kit (Pierce, Rockford, IL, United States). Equal amounts (20 μL) of protein were loaded onto the gels, and the separated proteins were transferred onto polyvinylidene fluoride membranes. The membranes were blocked in tris-buffered saline containing 5% Tween 20 and 5% skim milk at room temperature for 1 h and incubated overnight at 4°C using a primary antibody: MAPK, p-MAPK, ERK, p-ERK, p-mTOR, mTOR, and GAPDH (1:1,000; all antibodies were purchased from, Abcam, Cambridge, England). Primary antibodies were detected using a secondary anti-mouse IgG antibody coupled with horseradish peroxidase (HRP; 1:5,000, ab6728, Abcam, Cambridge, England). Target proteins were visualized using an EZ-ECL chemiluminescence detection kit (Pierce, Rockford, IL, United States).

### Immunohistochemistry

The brain tissue of the rats in each group was sliced, and protein expression was observed using immunohistochemistry. The sections were washed with phosphate buffered saline (PBS) and incubated overnight at 4 °C with primary antibody: MAPK, p-MAPK, ERK, p-ERK, p-mTOR, mTOR, and GAPDH (1:1,000; all antibodies were purchased from, Abcam, Cambridge, England). Subsequently, the sections were washed three times in PBS for 5 min and incubated with biotinylated goat anti-mouse immunoglobulin G (IgG) (1:1,000; ab150077; Abcam, China) at room temperature for 20 min. The sections were washed with PBS, and avidin-horseradish peroxidase complex was added (SABC Kit; Bost, Wuhan, China). The 3,3-diaminobenzidine (DAB) was used as a chromogen. The positive cells were visualized under a light microscope.

### Statistical analysis

Statistical analysis was performed using GraphPad Prism version 8.0 (GraphPad Software, Inc., San Diego, CA, United States). Between-group differences were compared using the unpaired Student’s *t* test. Multiple group comparisons were performed using analysis of variance (ANOVA) with Tukey’s *post hoc* test for pairwise comparison. The differences with *p* < 0.05 were considered to be statistically significant.

## Results

### Behavioral scores of rats

**Figure 1:**
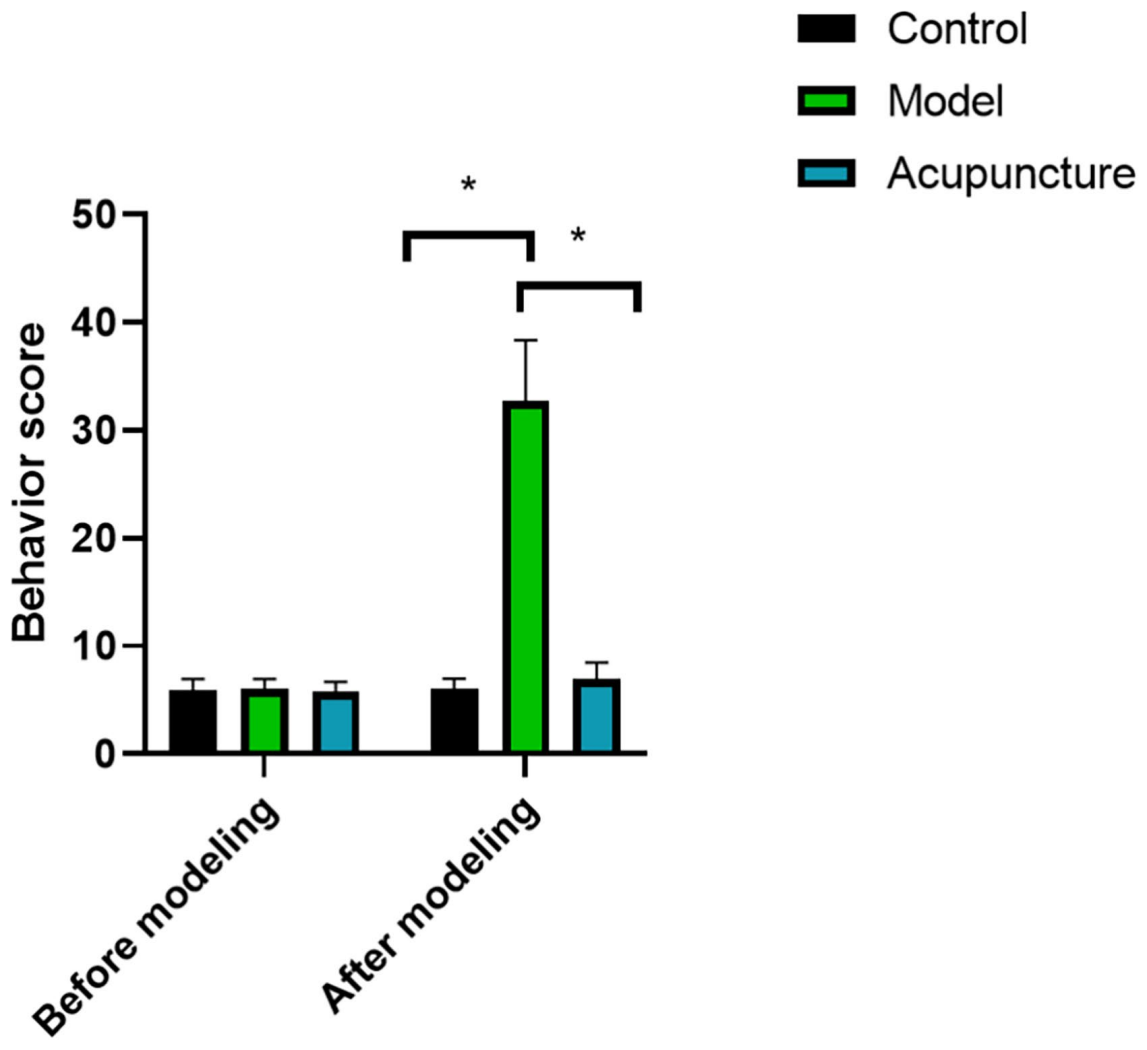
Comparison of behavior score in each group (*n* = 10 each). ^*^p < 0.05.

As shown in Figure 1, behavioral scores of rats in all groups had no statistical significance before modeling (*p* < 0.05). However, the behavioral score of the model group was significantly higher than that of the control group after modeling (*p* < 0.05), indicating that the animal model was successfully established. After acupuncture treatment, the behavioral score of rats was lower than that of model group, indicating that acupuncture treatment can reduce the behavioral activity of rats.

### Facial and hind-paw allodynia

**Figure 2:**
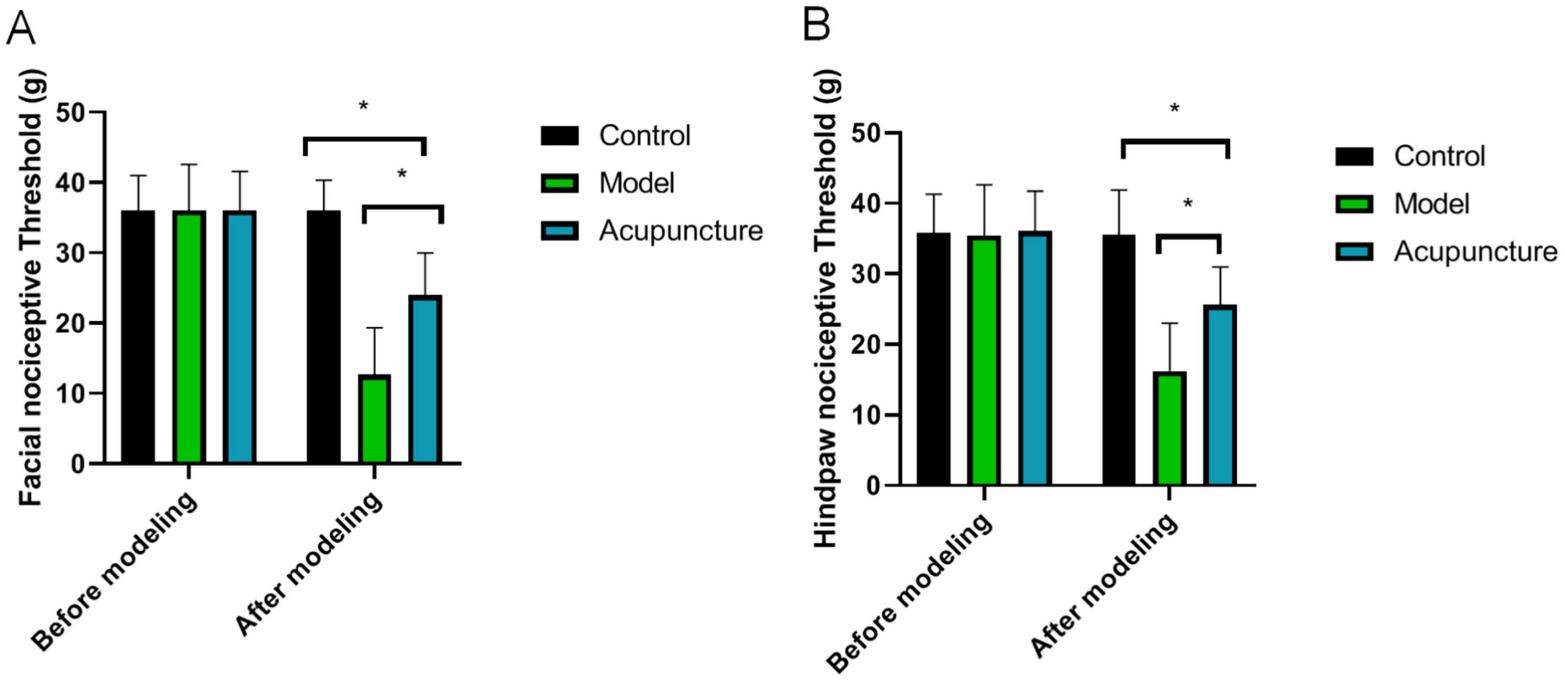
Comparison of facial and hind-paw nociceptive thresholds in each group (*n* = 10 each). **(A)** Facial allodynia, **(B)** Hind-paw allodynia. ^*^p < 0.05.

As shown in Figure 2, there was no significant difference in in facial or hind-paw nociceptive thresholds before modeling (*p* > 0.05). After modeling, the facial and hind-paw nociceptive thresholds in the model were significantly lower than that in the control group (*p* < 0.05), indicating that the animal models were successfully established. However, after acupuncture treatment, the facial and hind-paw nociceptive thresholds were higher than that of model group, indicating that acupuncture treatment have enhanced facial and hind-paw allodynia.

### Effects of acupuncture on expression levels of ET-1, IL-6, and TNF-*α*

**Figure 3:**
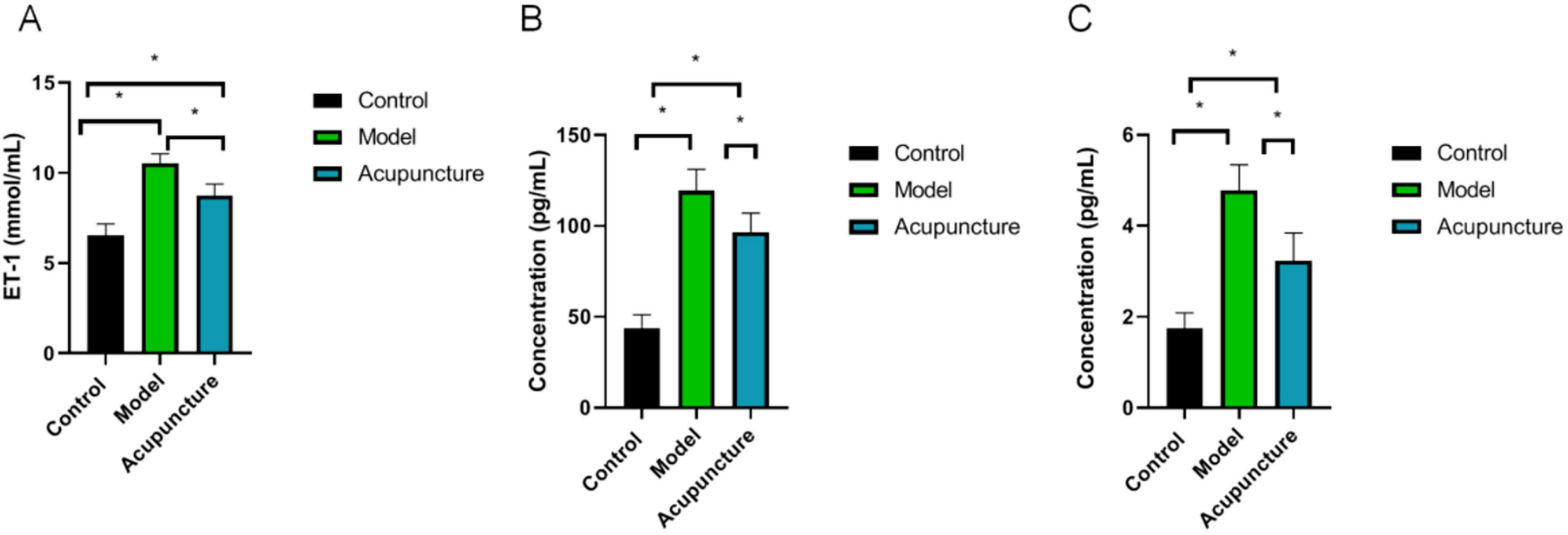
Plasma ET-1, interleukin (IL)-6 and tumor necrosis factor-alpha (TNF-*α*) levels in each group (*n* = 10 each). ET-1, IL-6 and TNF-*α* levels were determined using ELISA. **(A)** ET-1, **(B)** IL-6, **(C)** TNF-α. ^*^p < 0.05.

As shown in Figure 3, plasma ET-1, IL-6 and TNF-α levels in the model and acupuncture groups were significantly greater than those in the control group (*p* < 0.05). However, the levels of ET-1, IL-6 and TNF-α in the acupuncture group were lower than those in the model group (*p* < 0.05).

### Effect of acupuncture on CGRP mRNA expression

**Figure 4:**
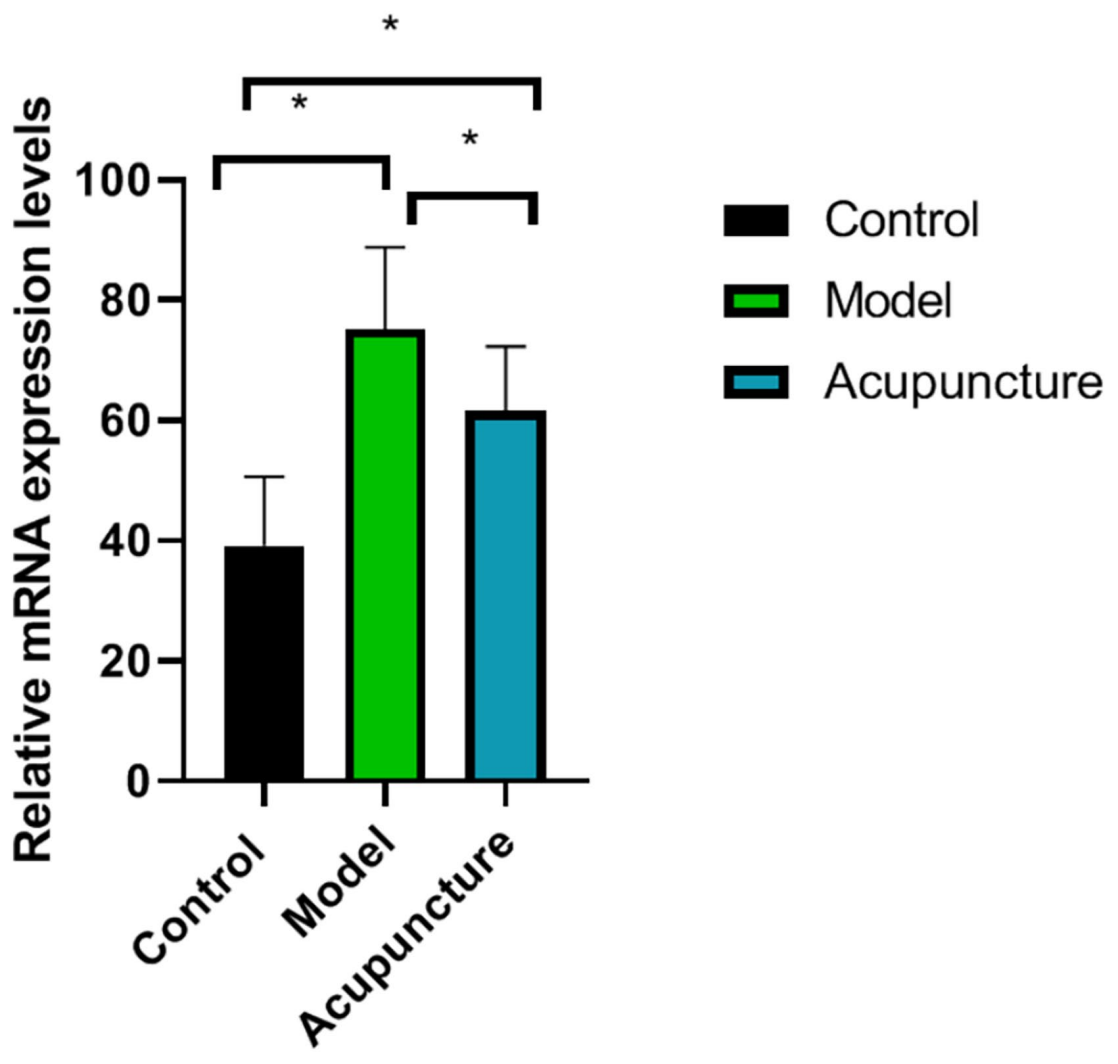
Comparison of calcitonin gene-related peptide (CGRP) messenger RNA expression in each group (*n* = 10 each). CGRP expression was determined using real-time quantitative polymerase chain reaction. ^*^p < 0.05.

Compared to the control group, CGRP mRNA expression in the model was significantly increased (*p* < 0.05). However, after acupuncture treatment, CGRP mRNA expression in the acupuncture group was significantly decreased (*p* < 0.05) (Figure 4).

### Effects of acupuncture on 5-HT levels in brain tissue

**Figure 5:**
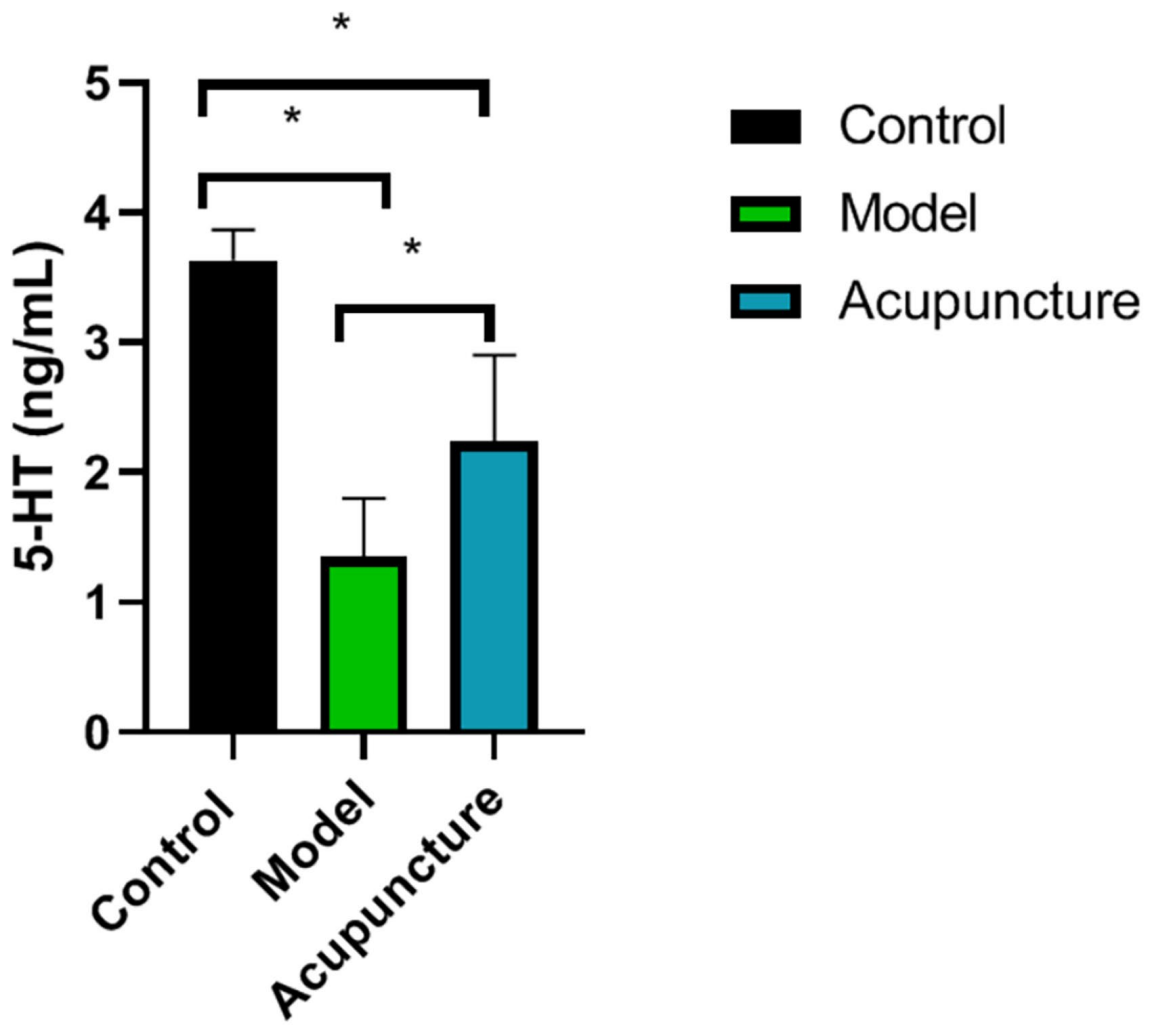
Comparison of 5-hydroxytryptamine (5-HT) levels in the brain tissue of rats in each group (*n* = 10 each). 5-HT levels were determined using ELISA. ^*^p < 0.05.

Compared with the control group, 5-HT levels in the model and acupuncture groups were significantly lower (*p* < 0.05). However, compared with the model group, 5-HT levels in the acupuncture group increased (*p* < 0.05) (Figure 5).

### Effect of acupuncture on expression level of ERK/MAPK pathway

**Figure 6:**
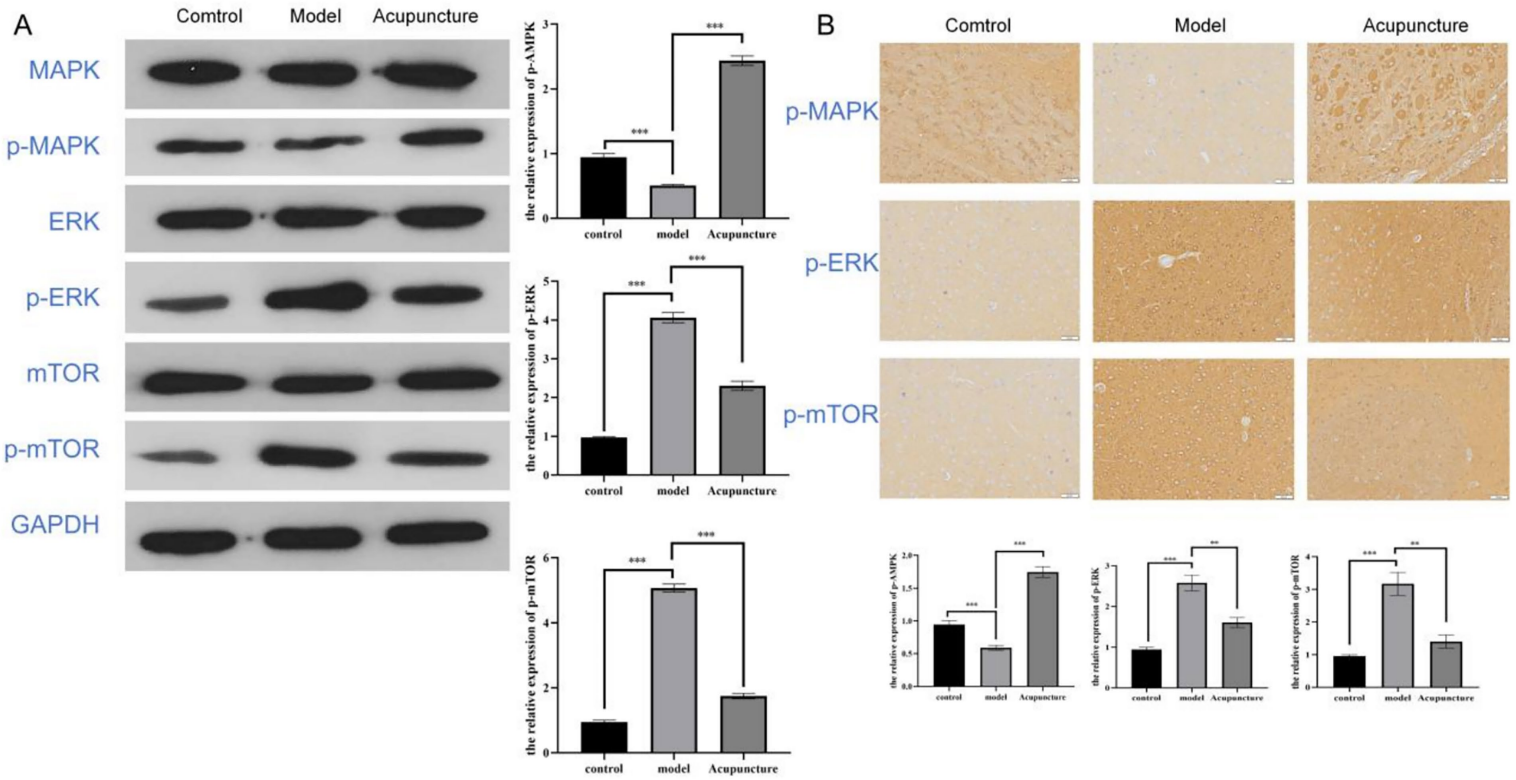
Acupuncture regulates ERK/MAPK pathway to relieve migraine. **(A)** Western blot analysis showing ERK/MAPK pathway protein expression levels in rats. **(B)** Immunohistochemical analysis showing ERK/MAPK pathway protein expression levels in rats at 200 × magnification. ^***^p < 0.001.

To further investigate the molecular mechanisms underlying these regulatory pathways, we determined whether acupuncture relieves migraine through the ERK/MAPK pathway. The protein levels of p-MAPK was decreased in the migraine rats, and the protein levels of p-ERK and p-mTOR were both increased. However, Acupuncture therapy reversed these changes (Figure 6A). Moreover, Immunohistochemical results showed that the expression changes of p-MAPK, p-ERK and p-mTOR in brain tissue of migraine rats were reversed after acupuncture treatment (Figure 6B).

## Discussion

Migraine is a kind of recurrent and often unilateral pulsing headache. The clinical pathogenesis of this disease is not clear, and it is generally believed that migraine is affected by multiple factors such as heredity and environment. Treatment includes both pharmacological and non-pharmacological interventions. Non-drug intervention can be through acupuncture or massage and other means to dilate blood vessels, relieve vasospasm, promote blood microcirculation. Migraine usually occurs on one or both sides of the head, and its associated pain is localized mostly in the Shaoyang meridians (21). It is closely related to the Shaoyang meridian; as such, the treatment should focus on dredging the Shaoyang meridian as the key acupoint selection of the hand and foot Shaoyang meridian, and Fengchi, Waiguan, and Yanglingquan as the main acupoints (22, 23). Acupuncture of Fengchi is claimed to have the effect of dispelling wind, relieving the surface, and propagating meridians (24). Based on the theory of TCM, *Waiguan* acupuncture can regulate “qi,” promote blood circulation, and relieve pain (25). Similarly it is considered to have the effect of dispelling wind and relieving the surface, dispersing evil and regulating “qi,” promoting blood circulation, and relieving pain (26). In addition, Yangling Spring activates the blood and dredging channels.

As treatment methods, warm needle acupuncture can make the veins of rats smooth, promote blood circulation and remove blood stasis, which can be used in the treatment of various diseases, including migraine, and have been widely used in some countries recently. The American Headache Association’s evidence-based guidelines for migraine has reported that warm needle acupuncture have potential benefits without side effects. Migraine has attracted great attention from the whole society. Although there are various methods to treat migraine, there are few studies on the mechanism of warm needle acupuncture on neurotransmitters, 5-HT and ET-1 in migraine rats.

The 5-HT is both a neurotransmitter and a pain-inducing substance, and participates in both pain-inducing and analgesic effects in the process of pain. 5-HT is an important neurotransmitter in the mechanism of acupuncture analgesia in the central system, which is secreted by cell bodies and dendritic axons located in the raphe nucleus of the brainstem medulla oblongata. 5-HT-ergic neurons in the brain are concentrated in the raphe nucleus group, which is an important structure for the modulation of pain sensation in the brain, thereby emitting ascending and descending 5-HT-ergic fibers. Acupuncture activate 5-HT ascending fibers, and inhibit the perception of nociceptive stimuli in the parathalamic tract. In addition, acupuncture also act on the brain structures related to nociceptive modulation and release 5-HT, which is helpful to enhance the analgesic effect. The descending 5-HT-ergic fibers can reach the posterior horn, lateral horn and anterior horn of the spinal cord along the dorsal lateral cord. When activated, 5-HT is released to inhibit the transmission of spinal cord nociceptive impulses in the way of presynaptic inhibition. The 5-HT ascending and descending pathways of raphe nucleus were selectively destroyed, and the analgesic effect of acupuncture was significantly weakened with the decrease of 5-HT content in the related brain.

In this study, 5-HT levels in the brain tissue of rats significantly increased after warm needle acupuncture. Studies have reported a link between plasma 5-HT levels and migraine (27). Abnormal levels of 5-HT, a strong vasoconstrictor circulating in the brain, can cause migraine (28). Plasma 5-HT has analgesic effects, and plasma 5-HT levels are lower than normal during migraine attacks (29). The increase in plasma 5-HT levels among patients with migraine is one factor explaining the increase in blood flow velocity caused by intracranial vasoconstriction. Warm needles can inhibit the mRNA expression of nitric oxide synthase in the brain stem and the trigeminal ganglion of rats, enhance the expression of 5-HT mRNA, regulate vasodilatation and contraction and, thus, exert an analgesic effect.

ET is a strong vasoconstrictor in the body (30). Platelet aggregation is strong in migraine rat model animals, and substances, such as ET-1, lead to vasoconstriction, which leads to increased bradykinin levels, blood circulation in the skull, and stasis in the head, resulting in migraine (31). Studies have shown that patients treated for migraine exhibit reduced blood viscosity, ET-1 levels, and vasodilation, which can improve the symptoms of head pain (32). Warm needle acupuncture can regulate the levels of endothelin and calcitonin-related peptides in plasma, regulate vasodilatation and contraction, improve brain microcirculation, and relieve migraine. In this study, plasma ET-1 levels in rats decreased after warm needle acupuncture, which was consistent with the results of the above study.

Currently, trigeminal neurovascular theory is the most convincing. According to this theory, the activation and sensitization of trigeminal neurovascular system (TGVS) is the pathophysiological basis of migraine, in which neurogenic inflammation is the key link. When trigeminal nerve endings distributed around the dural membrane and blood vessels are stimulated, they are released a variety of neuropeptides with inflammatory, painful and vasoactive effects were produced, which caused local neurogenic inflammation. In addition, the injury stimulation is uploaded along the trigeminal nerve to the trigeminal ridge nucleus of the brainstem of the secondary neuron, causing central sensitization, and then transmitted to the cerebral cortex through the thalamus of the tertiary neuron, resulting in headache. The activity level of trigeminal ridge nucleus neurons can reflect the degree of central sensitization to a certain extent.

In the present study, the level of CGRP mRNA in warm needle acupuncture-treated rats was lower than that in the model group. CGRP is a neurotransmitter and promoter of neurogenic inflammatory pain, which is widely distributed in the trigeminal spinal tract nuclei, trigeminal ganglia, and meningeal vessels of TGVS (33). CGRP can directly dilate blood vessels, degranulate mast cells, release inflammatory cytokines, such as IL-6, TNF-*α*, and IL-1, and promote the initiation and maintenance of peripheral sensitization of neurogenic inflammation (34). In addition, by activating the secondary neurons of the TGVS, pain signals can be locally expanded and transmitted to the thalamus and cerebral cortex, thereby activating and sensitizing the TGVS (35).

**Figure 7:**
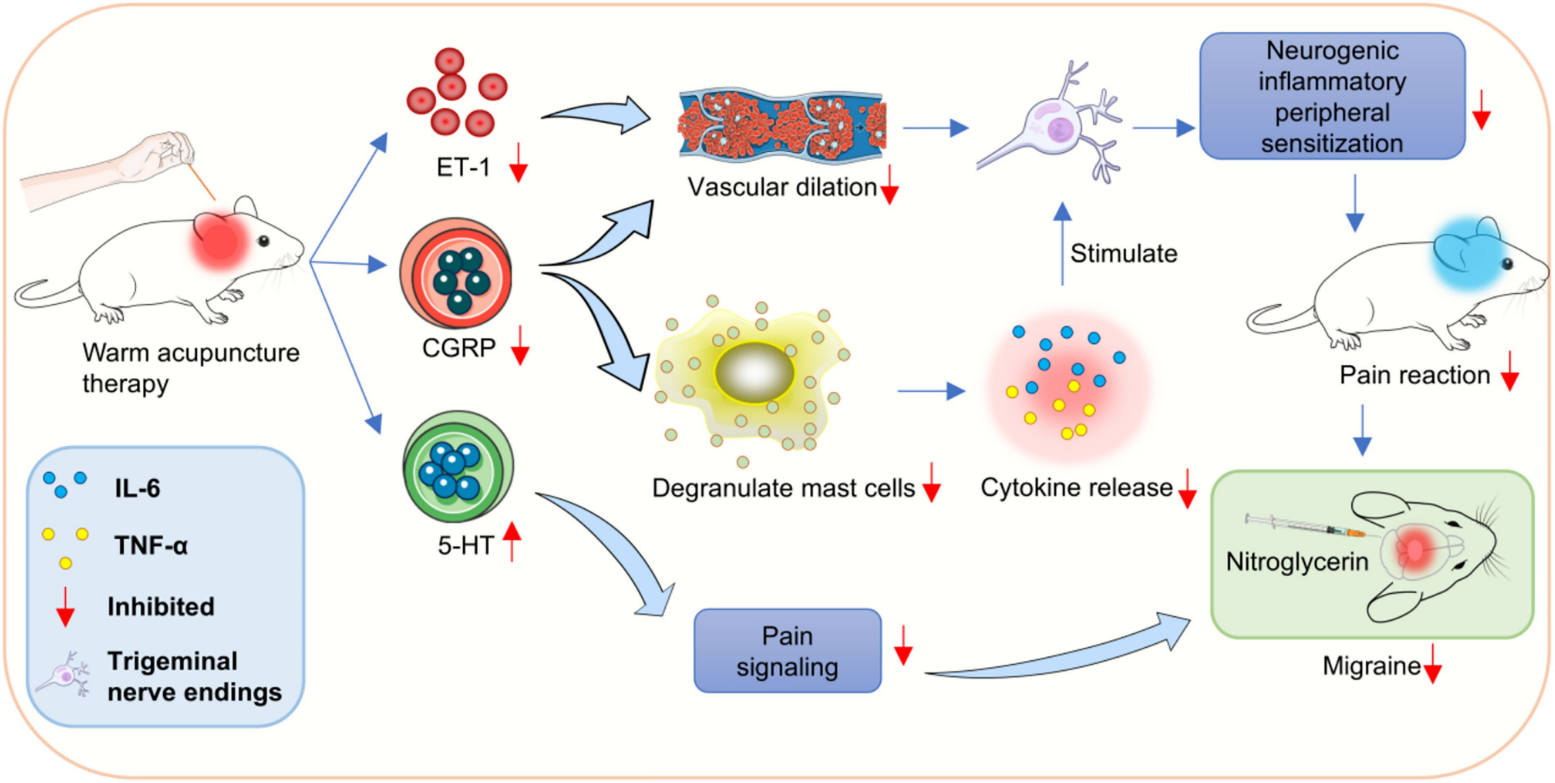
Possible molecular mechanism of warming acupuncture for relieving migraine in rats.

IL-6 and TNF-*α* are key inflammatory mediators and potential pain mediators and are mainly produced by neuronal microglia and astrocytes in the nervous system (36). IL-6 enhances the excitability of the meningeal afferent system, promotes pain signal transduction, and contributes to the occurrence of migraine. TNF-*α* can enhance the sensitivity of meningeal nociceptors by promoting the synthesis and secretion of inflammatory factors, such as CGRP prostaglandin, and the overexcitation of neurons, resulting in persistent head pain. We found that IL-6 and TNF-α induced pain sensitization in rats in a dose-dependent manner when administered to the dura mater or peripheral vessels. IL-6 and TNF-α levels are higher in migraine patients than in the normal population. In this study, the expression of IL-6 and TNF-α decreased after acupuncture, suggesting that acupuncture of Fengchi, Waiguan, and Yanglingquan can reduce the release of inflammatory factors, alleviate local neurogenic inflammation, inhibit the transmission of pain signals to the central nervous system, and relieve migraine in rats (Figure 7).

The study has some limitations. First, this study shows that warm needle acupuncture can relieve pain behavior in migraine model rats, but the optimal treatment time needs to be further determined. Increasing the sample size of the experimental mice and extending the treatment duration are also necessary for further verification of the therapeutic effect. Whether multiple treatments are more effective will also be the next direction of research. In addition, there is a high prevalence of migraine in adolescents. Whether warm needle acupuncture can have the same therapeutic effect in migraine rats of different ages will also be further studied. Moreover, although we found changes in IL-6, TNF-*α* and CGRP, there was a lack of effect size and power analysis, which will be validated in subsequent studies with larger samples. And subsequent studies will need to conduct a receptor-specific analysis of the 5-HT and its mechanism research. Furthermore, we only used male rats in this study, considering that females are usually affected by hormonal fluctuations and are more affected by migraines. So in future studies we need to pay attention to two kinds of sex selection. The applicability of warm needle acupuncture in human migraine patients was not demonstrated in this study. Therefore, we need to evaluate the clinical effects of warm needle acupuncture in human patients with migraine in follow-up studies. And, migraine involves multiple interactions between the neurovascular system and the nervous system, and further evaluation of the treatment effect of this study is needed compared to other models.

In conclusion, warm needle acupuncture of Fengchi, Waiguan, and Yanglingquan relieved pain in rats with migraine and reduced the expression of IL-6, TNF-*α*, 5-HT, ET-1, and CGRP. Moreover, its effect may be related to the MAPK/ERK pathway. It could be a potentially effective treatment for migraines.

### Preface paragraph

The Fengchi (GB20) is regarded as a major point for migraine treatment in acupuncture therapy (37); The “Baihui (GV20)” point is located on the top of the head and is connected to the brain, so it can awaken the brain and open the mind (38). The Fengfu (GV16) is one of the 13 ghost acupoints, an acupuncture prescription for mental illness, and both belong to the Governor Vessel (39). According to traditional Chinese medicine, both Hegu (LI4) is known as hubs for internal and external energy gathering and transforming. And the combination stimulus of Hegu (LI4) and Taichong (LR3) is defined as “the four gates,” which is usually applied to promote the circulation of “Qi” and blood throughout the whole body (40). Additional, because acupuncture at *Waiguan* (SJ5) has been shown to effectively improve sequelae of stroke such as hemiplegia and sensory disturbance, it is commonly selected as a target point for research (41).

## Data Availability

The original contributions presented in the study are included in the article/supplementary material, further inquiries can be directed to the corresponding authors.
